# Anisotropy in the magnetic interaction and lattice-orbital coupling of single crystal Ni_3_TeO_6_

**DOI:** 10.1038/s41598-018-33976-w

**Published:** 2018-10-25

**Authors:** Anirudha Ghosh, K.-H. Chen, X.-S. Qiu, S. H. Hsieh, Y. C. Shao, C. H. Du, H. T. Wang, Y. Y. Chin, J. W. Chiou, Sekhar C. Ray, H. M. Tsai, C. W. Pao, H. J. Lin, J. F. Lee, Raman Sankar, F. C. Chou, W. F. Pong

**Affiliations:** 1 Department of Physics, Tamkang University, Tamsui, 251 Taiwan; 2 Department of Physics, National Tsinghua University, Hsinchu, 300 Taiwan; 3 Department of Physics, National Chung Cheng University, Chiayi, 621 Taiwan; 4 Department of Applied Physics, National University of Kaohsiung, Kaohsiung, 811 Taiwan; 5 Department of Physics, University of South Africa, Johannesburg, 1710 South Africa; 6 National Synchrotron Radiation Research Center, Hsinchu, 300 Taiwan; 7 Center for Condensed Matter Science, National Taiwan University, Taipei, 106 Taiwan

## Abstract

This investigation reports on anisotropy in the magnetic interaction, lattice-orbital coupling and degree of phonon softening in single crystal Ni_3_TeO_6_ (NTO) using temperature- and polarization-dependent X-ray absorption spectroscopic techniques. The magnetic field-cooled and zero-field-cooled measurements and temperature-dependent Ni *L*_3,2_-edge X-ray magnetic circular dichroism spectra of NTO reveal a weak Ni-Ni ferromagnetic interaction close to ~60 K (*T*_SO_: temperature of the onset of spin ordering) with a net alignment of Ni spins (the uncompensated components of the Ni moments) along the crystallographic *c*-axis, which is absent from the *ab*-plane. Below the Néel temperature, *T*_N_~ 52 K, NTO is stable in the antiferromagnetic state with its spin axis parallel to the *c*-axis. The Ni *L*_3,2_-edge X-ray linear dichroism results indicate that above *T*_SO_, the Ni 3*d e*_g_ electrons preferentially occupy the out-of-plane 3*d*_3z_^2^_−r_^2^ orbitals and switch to the in-plane 3*d*_x_^2^_−y_^2^ orbitals below *T*_SO_. The inherent distortion of the NiO_6_ octahedra and anisotropic nearest-neighbor Ni-O bond lengths between the *c*-axis and the *ab*-plane of NTO, followed by anomalous Debye-Waller factors and orbital-lattice in conjunction with spin-phonon couplings, stabilize the occupied out-of-plane (3*d*_3z_^2^_−r_^2^) and in-plane (3*d*_x_^2^_−y_^2^) Ni *e*_g_ orbitals above and below *T*_SO_, respectively.

## Introduction

**Figure 1:**
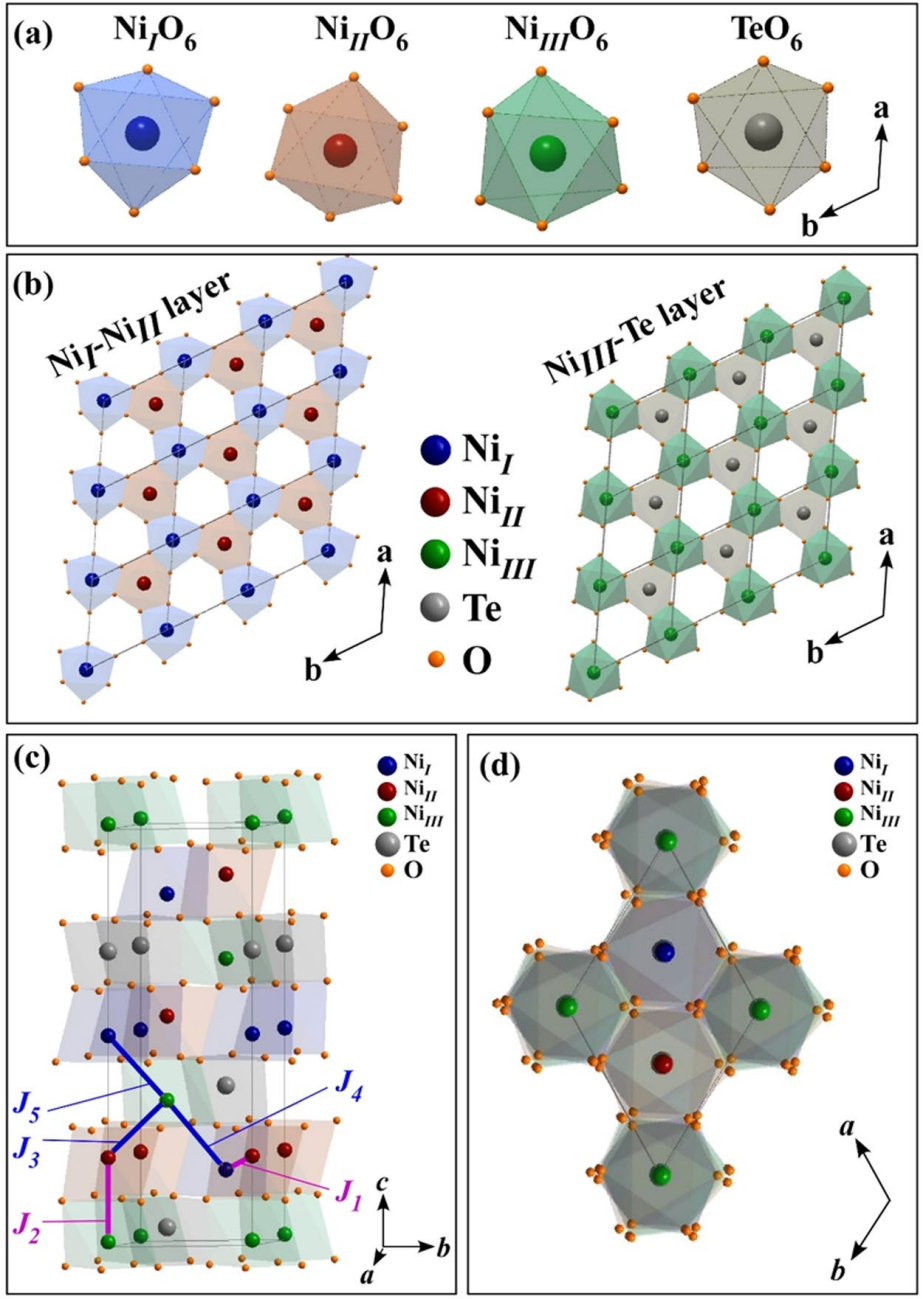
(**a**) Two-dimensional projection of Ni_*I*_O_6_, Ni_*II*_O_6_, Ni_*III*_O_6_ and TeO_6_ octahedra onto the ***ab***-plane. (**b**) Ni_*I*_O_6_-Ni_*II*_O_6_ and Ni_*III*_O_6_-TeO_6_ honeycomb-ring layers in the ***ab***-plane, stacked to form an NTO crystal structure with *R*3 lattice symmetry. (**c**) NTO crystal structure with magnetic exchange constants (*J*_1_*-J*_5_) among the Ni ions along various paths in the Ni_*I*_-Ni_*II*_ and Ni_*III*_-Te planes. (**d**) A different view of the NTO crystal structure in the ***ab***-plane.

Interest in 3*d* transition metal tellurates, *M*_3_TeO_6_ (*M* = 3*d* transition metals), has been rising since the beginning of this century owing to their rich crystalline and multiferroic properties^1–4^; they also offer unprecedented opportunities for the development of spintronics and information storage devices^5,6^. One of the members of this class of tellurates is Ni_3_TeO_6_ (NTO), which was developed approximately half a century ago as a result of the efforts of Newnham and Meagher^7^, who were searching for a multifunctional dielectric-magnetic material. However, the physical properties of this system received little research attention until 2006^8,9^, when Becker and Berger^10^ studied its crystal structure and reignited interest in the material. NTO has a noncentrosymmetric rhombohedral (trigonal crystal structure, space group *R3*) lattice with both chirality and polarity. It has three crystallographically inequivalent Ni sites (Ni_*I*_, Ni_*II*_ and Ni_*III*_), which can be thought of as comprising a superstructure of α-Al_2_O_3_^11^. In NTO, the cation octahedra form Ni_*I*_O_6_-Ni_*II*_O_6_ and Ni_*III*_O_6_-TeO_6_ pairs by sharing the edges of octahedral faces [Fig. 1(a,b)]. The edge-sharing Ni_*I*_O_6_ and Ni_*II*_O_6_ octahedral pairs constitute a slightly corrugated, almost planar, honeycomb lattice, while the edge-sharing Ni_*III*_O_6_ and TeO_6_ octahedral pairs form an adjacent plane [Fig. 1(b)] with an offset of (***a***/3, ***a***/3) with respect to the honeycomb layer^12,13^. Figure 1(c,d) show different views of the crystal structure of NTO.

Based on spin-polarized density functional calculations, Wu *et al*.^14^ calculated the spin exchange constants (*J*_1_-*J*_5_) between the Ni sites in the NTO system. They calculated that *J*_1_ (for the exchange interaction between Ni_*II*_ and Ni_*I*_ in the ***ab***-plane) and *J*_2_ (for the exchange interaction between Ni_*II*_ and Ni_*III*_ along the ***c***-axis) are ferromagnetic (FM) and that *J*_2_ is much stronger than *J*_1_. In contrast, the exchange constants *J*_3_, *J*_4_ and *J*_5_ (for the exchange interactions Ni_*III*_-Ni_*II*_, Ni_*III*_-Ni_*I*_ and Ni_*I*_-Ni_*III*_, respectively) are antiferromagnetic (AFM). The difference between *J*_4_ and *J*_5_ derives from the variation in the Ni_*III*_ -O- Ni_*I*_ [Ni_*I*_ is in the bottom layer with respect to Ni_*III*_, as shown in Fig. 1(c)] and Ni_*I*_ -O- Ni_*III*_ [Ni_*I*_ is in the top layer with respect to Ni_*III*_, as shown in Fig. 1(c)] bond distances and bond angles, as mentioned in ref.^14^. Wu *et al*.^14^ also found that magnetic dipole-dipole interactions, which are stronger than spin-orbit coupling, are responsible for the orientation of the Ni spin axis parallel to the ***c***-axis. Figure 1(c) displays all these spin exchange paths. The unique noncentrosymmetric structure and variation in exchange interaction with various Ni spin sites together give rise to the favorable magnetic field-driven electric polarization properties of NTO^15–17^. Yokosuk *et al*.^15,16^ and Kim *et al*.^17^ used extremely high magnetic fields to elucidate the spin-induced electric polarization properties between 9 and 52 T. In a nominal magnetic field, NTO is a collinear antiferromagnet below ~52 K (Néel temperature: *T*_N_)^15^, as revealed by anisotropic magnetization and specific heat capacity measurements^12,13^. Yokosuk *et al*.^16^ and Skiadopoulou *et al*.^18^ performed a combination of infrared, Raman and THz spectroscopic measurements and explained the anomalous temperature-dependent behavior of spin excitation by spin-phonon coupling below *T*_N_, whereby local lattice distortion in the ***ab***-plane is induced by magnetic ordering, which subsequently modifies the AFM interaction between Ni ions owing to their displacements from their mean positions in the unit cell^16^. Moreover, numerous reports on related AFM systems have suggested a strong correlation among spin, orbital, charge and lattice degrees of freedom, which are responsible for the intriguing material properties of transition metal oxides^19–26^. Specifically, Ling *et al*.^19^ reported that a structural transition in lanthanum manganate can trigger Mn 3*d e*_g_ orbital ordering, causing AFM spin ordering. Deshpande *et al*.^20^ also found temperature- and substrate-driven preferential electron occupancy of the Mn 3*d e*_g_ orbital in La_0.85_Zr_0.15_MnO_3_ (LZMO) thin films epitaxially grown on SrTiO_3_ (STO) and MgO substrates. As the temperature is reduced from room temperature to below the magnetic transition temperature, the preferred orbital of the Mn 3*d e*_g_ state changes from the in-plane 3*d*_*x*_^2^_−*y*_^2^ to the out-of-plane *d*_3*z*_^2^_−*r*_^2^ orbital for LZMO/STO and vice versa for LZMO/MgO owing to the nature of the strain between the epitaxial film and the substrate. Experimental results further suggested that the strong tensile strain stabilizes the 3*d*_*x*_^2^_−*y*_^2^ orbital by inducing lattice distortions of the MnO_6_ octahedra in LZMO/MgO^20^. Furthermore, in *t*_2g_ systems such as rare-earth vanadate, cooperative orbital ordering induces local lattice distortion below a certain transition temperature^21^. Therefore, the origin of all of the aforementioned exotic properties of NTO is believed to be closely associated with the intriguing interplay between the Ni 3*d* electron’s spin, orbital, charge and lattice-related degrees of freedom, which could lead to intriguing magnetic properties, such as the existence of FM and AFM interactions with the spin-axis parallel to the crystallographic *c*-axis in NTO^27–30^. Although the spin orientations of Ni^12,14,16^ and the spin-phonon coupling in NTO^15,16,18^ have recently been studied, the spin-orbit-lattice-charge intercorrelations have not yet been fully explored, to the best of our knowledge. Therefore, this investigation is a detailed study of the temperature- and polarization-dependent electronic and atomic structure of NTO, as well as of the preferential orbital and anisotropic magnetic behaviors, to elucidate correlations among the aforementioned degrees of freedom across the transition temperature of the NTO single crystal.

## Results and Discussion

**Figure 2:**
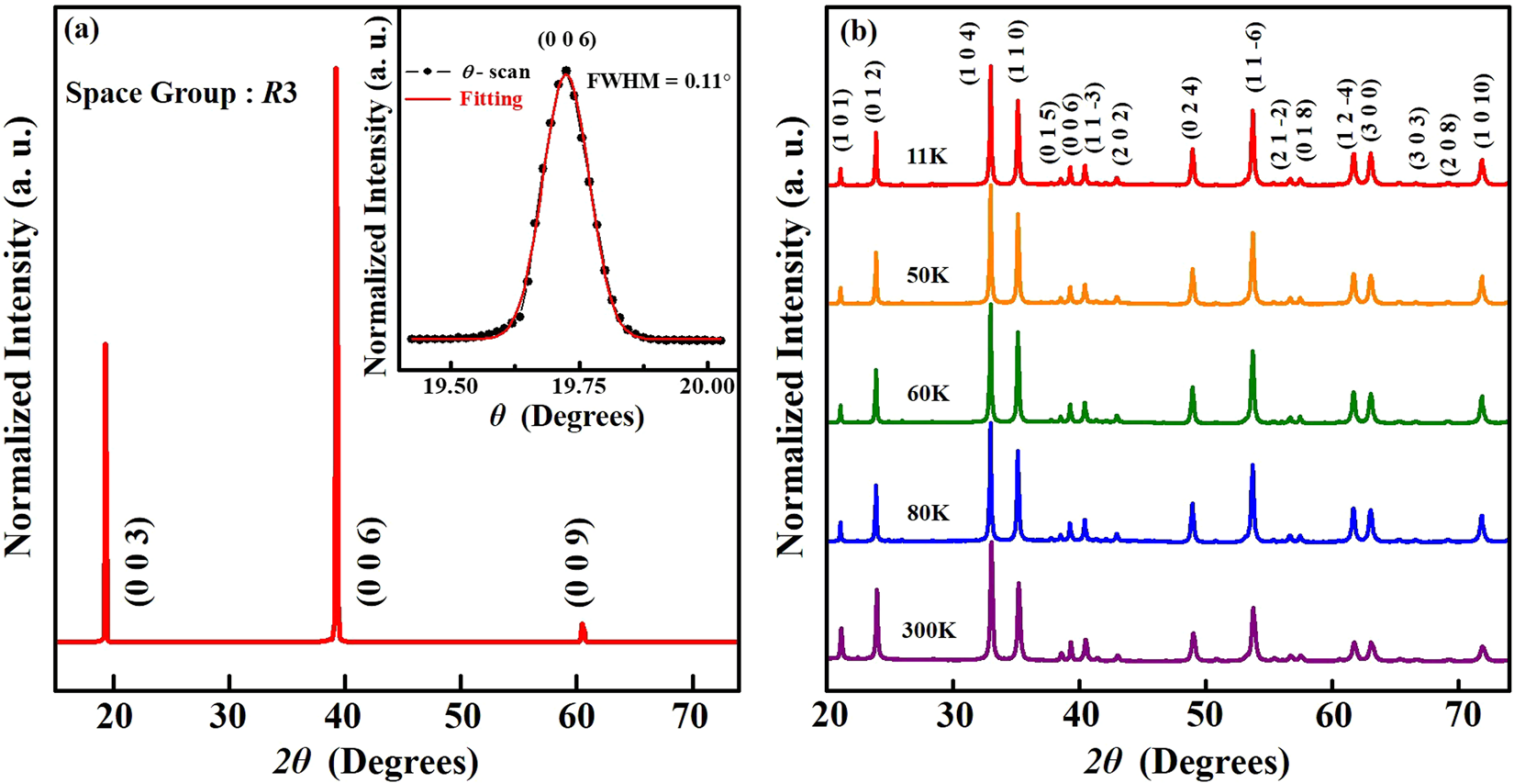
(**a**) 2*θ* single-crystal XRD plot on the (003) plane of NTO. The inset shows the *θ* scan that corresponds to the (006) Bragg peak, which clearly reveals the single crystalline phase of NTO with a single Gaussian peak and a narrow FWHM feature. (**b**) Temperature-dependent powder XRD plot, measured after finely crushing a few NTO crystals. Details of all the peak positions and corresponding (*hkl*) values can be found in Table 1 of the Supplementary Information.

Figure 2(a) displays the X-ray diffraction (XRD) pattern of the NTO single crystal at room temperature, showing the [003] Bragg reflection. The diffraction line shape is symmetrical, and Gaussian fitting yields a small full-width-at-half-maximum (FWHM = 0.11^0^), as observed in the *θ* scan of the (006) Bragg reflection in the inset of Fig. 2(a). This very small FWHM indicates good crystallinity and chemical homogeneity of the NTO single crystal. Figure 2(b) also shows the temperature-dependent XRD pattern (all indexed peaks^7^ are tabulated in Table 1 of the Supplementary Information) of crushed and finely ground NTO powder obtained from its single crystal. All data are similar at all temperatures of interest with no appearance or disappearance of peaks, which shows that NTO exhibits no structural phase transition in the investigated temperature range of 11–300 K. However, the intensities of some peaks [such as (1 0 1), (0 1 2) and (1 1 0)] relative to the most intense (1 0 4) peak vary with temperature; the implications of this finding will be discussed later in this manuscript. Sankar *et al*.^13^ conducted XRD analyses of NTO using the general structure analysis system code^31^ and identified a trigonal crystal structure in a rhombohedral lattice with space group *R*3 and cell parameters ***a*** = ***b*** = 5.11 Å and ***c*** = 13.74 Å at 300 K.

**Figure 3:**
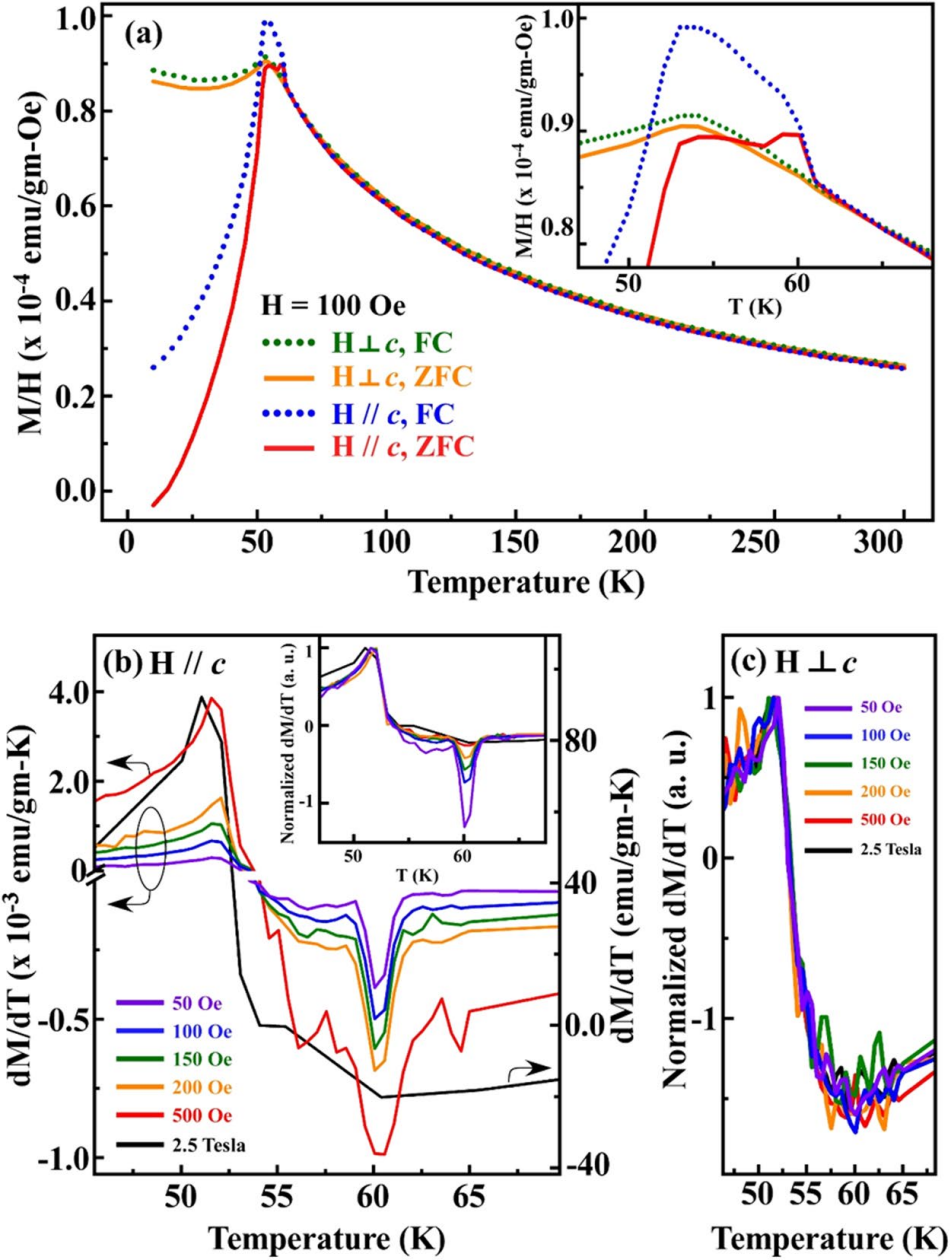
(**a**) Magnetic susceptibility (M/H) vs. temperature plots for the NTO single crystal under an external magnetic field of H = 100 Oe, applied parallel and perpendicular to the crystallographic ***c***-axis. The inset shows an expanded view of the 47–67 K region, which includes a weak shoulder-like feature close to *T*_SO_ (~60 K) in both FC and ZFC modes when the magnetic field is applied parallel to the ***c***-axis. (**b**) Derivative of magnetization (M) versus temperature (T) plots for H// ***c*** at various magnetic fields to provide a better view of the feature close to *T*_SO_. The dM/dT plot at the 2.5 T magnetic field correspond to the right axis, whereas the dM/dT plots at weaker magnetic fields correspond to the left axis, as shown by the arrows. The inset shows the corresponding *normalized* dM/dT plots [(dM/dT)/(dM/dT at T = *T*_N_)]. (**c**) *Normalized* dM/dT plots for H⊥ ***c***.

Figure 3(a) plots the magnetic susceptibility (M/H) as a function of temperature [in the field-cooling (FC) and zero-field-cooling (ZFC) modes] using a nominal external magnetic field H = 100 Oe aligned parallel and perpendicular to the ***c***-axis, thus revealing the anisotropic magnetic properties of NTO. The FM interaction [mostly a result of a *J*_2_ exchange interaction, as depicted in Fig. 1(c)] is rather weak and appears as a hump close to ~60 K (*T*_SO_: spin-ordering temperature) when the magnetic field is applied parallel to the ***c***-axis [inset in Fig. 3(a)] and it is absent when the magnetic field is applied perpendicular to the ***c***-axis (H⊥ ***c***). The weaker FM interaction suggests that the Ni spins may not be collinear. Instead, the uncompensated component of the Ni spin moment is aligned parallel to the ***c***-axis (hereafter referred to simply as FM Ni spins) and the interaction between these components of the moments will be referred to as FM interaction in this manuscript. Below ~52 K (*T*_N_), the M/H curves (for H// ***c*** and H⊥ ***c***) turn downward, revealing AFM ordering (caused by *J*_3_-*J*_5_ exchange interactions). The different magnetization features revealed by the H// ***c*** and H⊥ ***c*** curves below *T*_N_ (~52 K) suggest that the AFM spin axis is primarily parallel to the ***c***-axis^12^. This alignment of the magnetic spin axis parallel to the ***c***-axis in the FM and AFM phases is consistent with the earlier calculations of Wu *et al*.^14^. Sankar *et al*.^13^ claimed that in a high magnetic field, most of the Ni ions in NTO are in the Ni^3+^ state in either the high-spin (*S* = 3/2; *t*_2g_^5^*e*_g_^2^) or low-spin (*S* = 1/2; *t*_2g_^6^*e*_g_^1^) configuration, with the remaining minority in the Ni^2+^ state with the *S* = 1 (*t*_2g_^6^*e*_g_^2^) spin configuration. Their analysis reflects that at low magnetic fields, however, the Ni^2+^ spin state in NTO is responsible for magnetization. Temperature- and polarization-dependent Ni *K*-edge X-ray absorption near-edge structure (XANES) analyses of the NTO single crystal, as well as Ni *K*- and *L*_3,2_-edge XANES analyses of powdered NiO, Ni_2_O_3_ and NTO at room temperature, have also been performed, as described in Fig. S1 of the Supplementary Information. Consistent with the findings of Wiegart *et al*.^32^, the threshold positions of the Ni *K*-edge XANES of NTO and the reference samples NiO (Ni^2+^) and Ni_2_O_3_ (Ni^3+^)^33^, used for determining the valence state of the Ni ions, are similar. However, the areas under the Ni *K* pre-edge features (which originate due to unoccupied Ni *d*/*p-d* hybridized states and result in quadrupole Ni 1*s* → 3*d* and dipole allowed 1*s* → *pd* ligand-metal hybridized state transitions^34^ close to 8332–8333 eV in the Ni *K*-edge absorption spectra) differ substantially between the Ni^2+^ and Ni^3+^ states in NiO and Ni_2_O_3_, respectively [see the discussion in S1]. The Ni *L*_3,2_-edge XANES analyses of powdered NTO, NiO and Ni_2_O_3_ indicate that due to the larger number of unoccupied Ni 3*d* states in Ni_2_O_3_ compared to NiO, the areas under the Ni *L*_3,2_-edge (22.33 ± 0.05) [Fig. S1(c)] and *K* pre-edge (0.12 ± 0.02) features [insets in Fig. S1(a,b)] in Ni_2_O_3_ are higher than those in NiO (20.08 ± 0.05 and 0.05 ± 0.02 at the Ni *L*_3,2_- and *K*-edge, respectively). Clearly, for NTO, the areas under the Ni *L*_3,2_-edge (21.05 ± 0.05) and *K* pre-edge (0.05 ± 0.01 and 0.06 ± 0.01 for ***E***// ***c*** and ***E***⊥ ***c***, respectively) features are close to those for NiO, suggesting that most of the Ni ions in NTO are in the 2 + valence state. Additionally, the Ni *L*_3,2_-edge absorption line shapes of NTO are consistent with those of NiO in the results of Hu *et al*.^35^ and Abbate *et al*.^36^, who compared the Ni *L*_3,2_-edge absorption line shapes of NiO with those of other Ni compounds to estimate the valence states. To determine the valence state of the Ni ions in NTO at various temperatures, as shown in the bottom panels of Fig. S1(a,b) based on the position of the threshold feature, the derivative of the threshold feature of the Ni *K*-edge absorption feature is used. Apparently, the Ni *K*-edge threshold energy and the line shapes of the NTO single crystal, as well as the area under the pre-edge peak, do not vary with temperature for two electric polarizations (***E***// ***c*** and ***E***⊥ ***c***), confirming that the valence state of the Ni ions is insensitive to temperature and orientation and is mostly 2+ in NTO. Furthermore, we have also carried out temperature-dependent Te *K*-edge XANES analyses [Fig. S2(a,b) for ***E***// ***c*** and ***E***⊥ ***c***, respectively] as complementary evidence to support the Ni^2+^ state. The threshold/peak position of the Te *K*-edge XANES is 31825.0 ± 0.5 eV for both the ***E***// ***c*** and ***E***⊥ ***c*** polarizations, which is consistent with the XANES spectra for the Te^6+^ state as reported by Grundler *et* *al*.^37^ for their Te(OH)_6_ sample. Assuming oxygen in the O^2−^ state, the 6+ valence state of Te evidently suggests that Ni will be in the 2+ valence state to satisfy charge compensation in NTO. Moreover, similar to the Ni *K*-edge XANES, the Te *K*-edge XANES is also insensitive to changes in temperature within the measured 40–300 K range. This result further indicates that Te and Ni are stable in their respective 6+ and 2+ states and do not vary with temperature within this range. Evidently, the temperature- and polarization-dependent Ni *K*-edge XANES studies do not support any substantial effect of the disproportionation of Ni^3+^ and Ni^2+^ on the anisotropic magnetic properties of NTO below the transition temperature *T*_N_ because the Ni ions in NTO are primarily not in the Ni^3+^ state and therefore do not exhibit high-spin or low-spin configurations at various temperatures.

In the FC and ZFC curves of NTO for H// ***c*** in Fig. 3(a), the FM region is barely observable relative to the dominant AFM feature in NTO. Therefore, for better understanding of the FM feature in NTO, magnetization measurements were performed in various magnetic fields, and the first derivatives (dM/dT) for H// ***c*** and H⊥ ***c*** are plotted in Fig. 3(b,c), respectively. dM/dT is useful in the sense that it can be used to identify minor features or fluctuations of magnetization with temperature, which are difficult to observe from raw data^38,39^. Although the measured absolute intensity of the FM feature for H// ***c*** gradually increases with the magnetic field, as shown of Fig. 3(b), the corresponding *normalized* dM/dT plots [*normalized* dM/dT = (dM/dT)/(dM/dT at *T*_N_)] in NTO gradually decrease, as shown in the inset in Fig. 3(b). The *normalized* dM/dT plots enable the identification of the variation in the FM feature with respect to the AFM feature in NTO under various magnetic fields. The plots in the inset of Fig. 3(b) indicate that in a stronger magnetic field, the AFM feature dominates the FM feature and since they are close to each other on the temperature scale, the FM feature is generally invisible. This fact may explain why the results of Sankar *et al*.^13^ indicate several different magnetic behaviors of NTO in high and low magnetic fields. In contrast, the field-dependent *normalized* dM/dT plots for H⊥ ***c*** do not show any features close to ~60 K, as presented in Fig. 3(c), suggesting a lack of alignment among the FM Ni spins of NTO in the ***ab***-plane. To further understand the correlation of the anisotropic FM and AFM phases with the preferential orbital and lattice degrees of freedom in the NTO single crystal, temperature-dependent X-ray magnetic circular dichroism (XMCD), X-ray linear dichroism (XLD) and extended X-ray absorption fine structure (EXAFS) analyses were performed and are described below.

**Figure 4:**
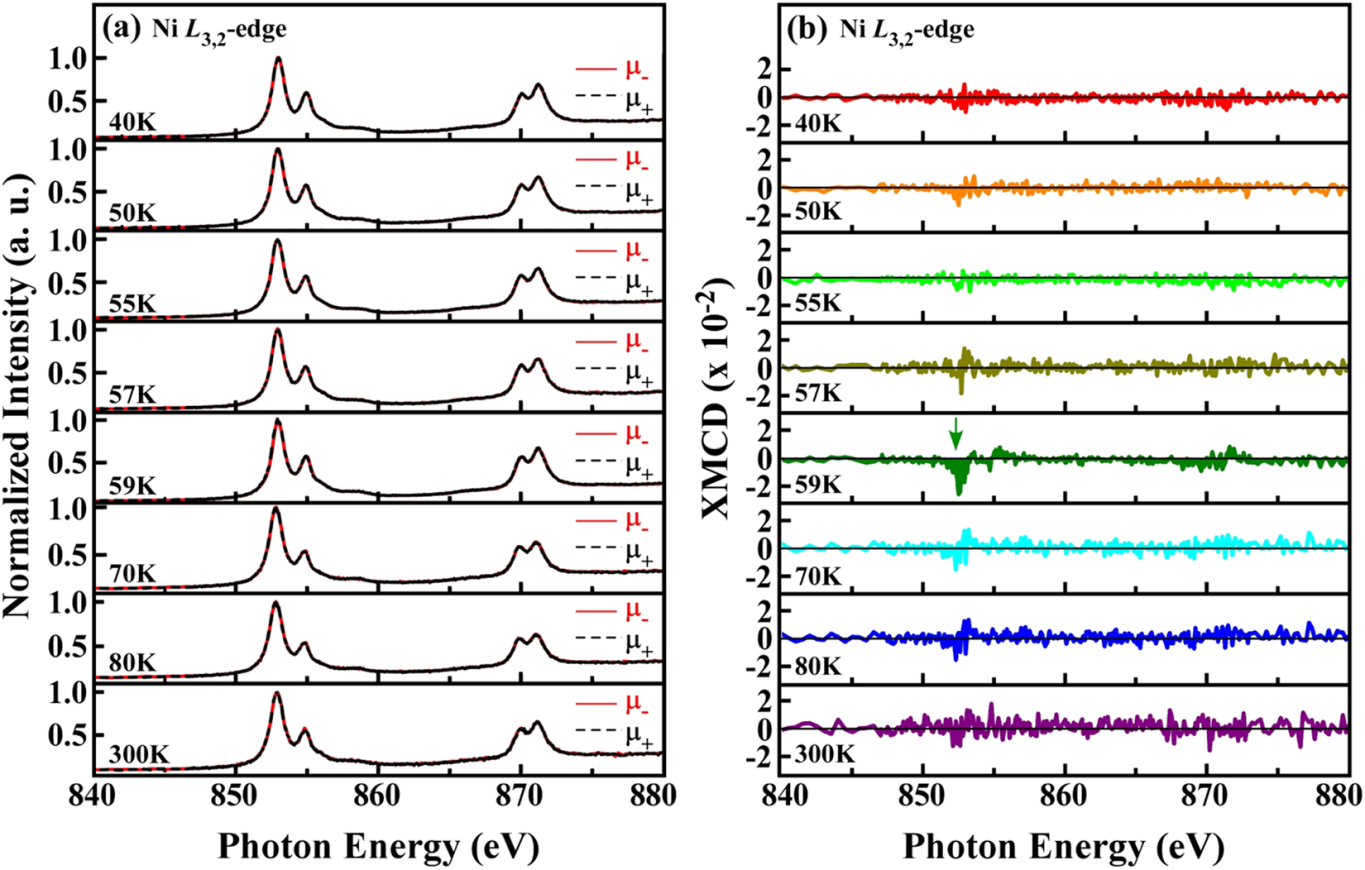
(**a**) Temperature-dependent X-ray absorption spectra (with circularly polarized X-ray photons) of single crystal NTO at the Ni *L*_3,2_-edge in a magnetic field (100 Oe) applied parallel (μ_+_) and antiparallel (μ_−_) to the ***c***-axis of NTO, and (**b**) corresponding XMCD spectra, calculated from the difference in the X-ray absorption spectra obtained at the magnetic fields in two directions [(μ_−_ − μ_+_)/(μ_−_ + μ_+_)]. The prominent (negative) XMCD signal at the Ni *L*_3_-edge (indicated by the green arrow) at 59 K reveals the existence of FM interaction with Ni spins parallel to the ***c***-axis in NTO.

Figure 4(a) displays the temperature-dependent Ni *L*_3,2_-edge XANES spectra of the NTO single crystal, with the photohelicity of incident X-rays parallel (μ_+_) and antiparallel (μ_−_) to the direction of magnetization under a magnetic field of H = 100 Oe applied parallel and antiparallel to the ***c***-axis, respectively. All temperature-dependent Ni *L*_3,2_-edge XANES spectra of NTO exhibit two broad features in the ranges of 852–857 eV and 868–873 eV, which are attributed to Ni 2*p*_3/2_ → 3*d*_5/2_ and 2*p*_1/2_ → 3*d*_3/2_ dipole transitions, respectively. Figure 4(b) also shows the corresponding Ni *L*_3,2_-edge XMCD spectra [(μ_−_ − μ_+_)/(μ_−_ + μ_+_)] obtained at various temperatures. From the field-dependent *normalized* dM/dT in the inset of Fig. 3(b), the FM feature gradually becomes obscured by the AFM feature at higher magnetic fields. Therefore, a considerable XMCD signal from NTO can be obtained at an applied magnetic field of 100 Oe without being buried under the dominating AFM feature. XMCD reveals the expectation value of the magnetic moment, <*M*>^40,41^, and thereby provides information about individual magnetic ion spins and magnetic orbital moments in the overall FM interactions^42,43^. Clearly, the temperature-dependent Ni *L*_3,2_-edge XMCD spectra obtained by switching the magnetic field from parallel to antiparallel to the ***c***-axis reveal a weak but clear XMCD signal at 59 K [close to *T*_SO_ (60 K)_,_ marked by a green arrow in Fig. 4(b)] that diminishes as temperature varies upward or downward. The directionality of the FM spins of the Ni ions along the ***c***-axis in NTO is consistent with the magnetization measurements herein (Fig. 3) and in other studies^12,13^. Importantly, in contrast, the temperature-dependent Ni *L*_3,2_-edge XMCD measurements made when a magnetic field of 100 Oe is applied perpendicular to the ***c***-axis (two opposite magnetic field directions) do not reveal an XMCD signal (see Fig. S3 in the Supplementary Information). In magnetic materials, the FM moment is typically correlated with the degree of the long-range magnetic order of the magnetic ions, which is determined by competitive exchange coupling between electron spins and thermal fluctuation. However, the XMCD measurements in Fig. 4(b) clearly demonstrate weak FM interaction between the Ni 3*d* electron spins in NTO at ~59 K with the magnetic field along the ***c***-axis.

**Figure 5:**
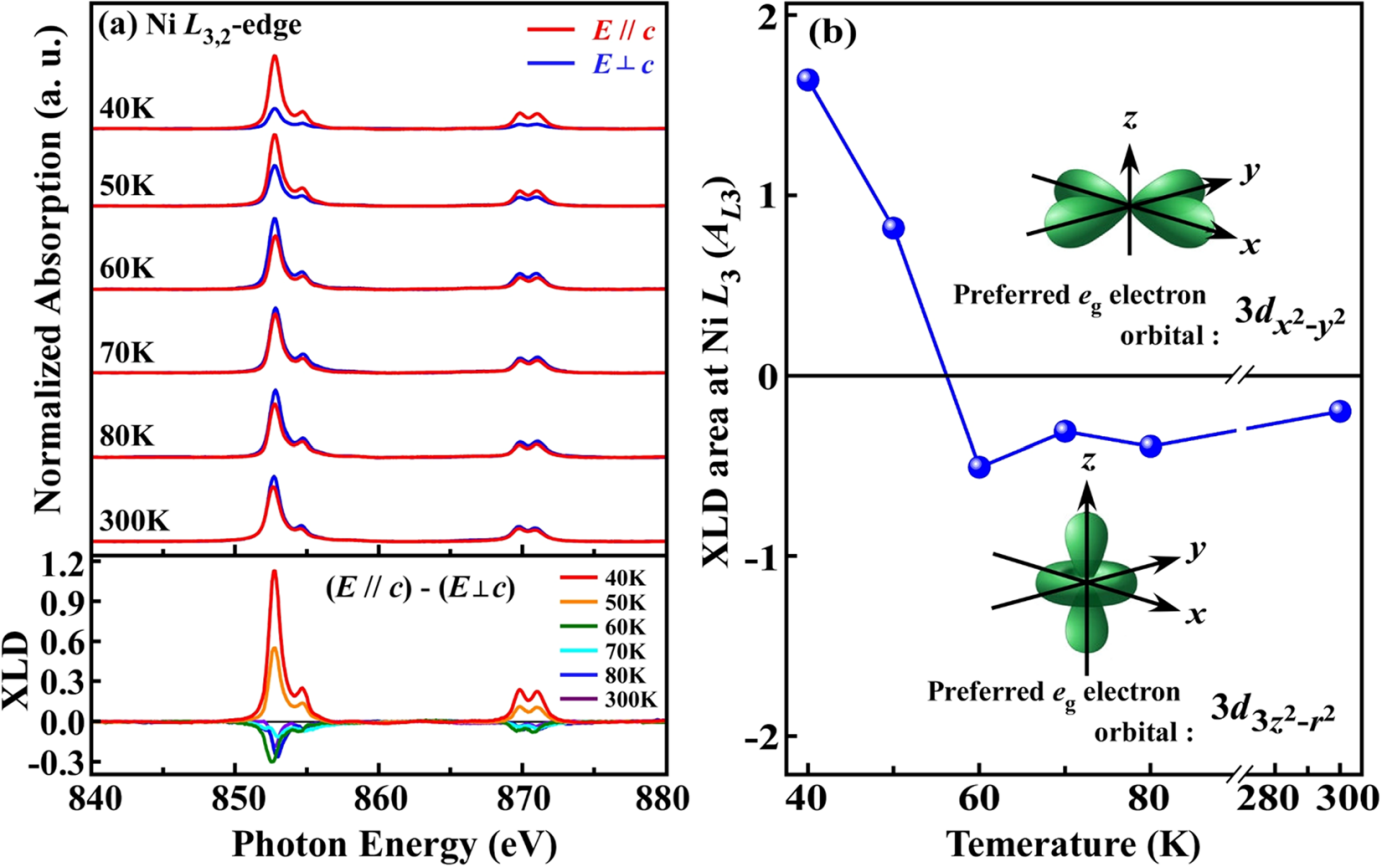
(**a**) Temperature-dependent Ni *L*_3,2_-edge X-ray absorption spectra obtained using linearly polarized X-rays with ***E***// ***c*** and ***E***⊥ ***c*** polarizations, with the corresponding XLD [(***E***// ***c***) − (***E***⊥ ***c***)] shown at the bottom. (**b**) Temperature-dependent variation in the XLD area (integrated over the range of 847–857 eV of the XLD spectra) at the *L*_3_-edge (*A*_*L*3_), shown to elucidate the thermal evolution of the preferential electron occupancy in the Ni 3*d e*_g_ orbital, which switches from in-plane (3*d*_x_^2^_−y_^2^) to out-of-plane (3*d*_3z_^2^_−r_^2^) above *T*_SO_ (60 K).

Typically, XMCD is sensitive only to the expectation value of the local magnetic moment, <*M*>, and therefore disappears in the AFM regime^40,41^ at or below *T*_N_ ~ 52 K, as shown in Fig. 4(b). The simultaneous determination of the spin orientation in an AFM system, unlike in an FM system, and local orbital structure is a challenging experimental task because of their compensated magnetic dichroism nature. However, with the development of synchrotron sources with high photon fluxes, soft XLD spectroscopy has emerged as a powerful tool for detecting the spin axis and orbital symmetry of all uniaxial magnetic systems because linearly polarized photons have only axiality, which favors the measurement of the expectation value of the square of the magnetic moment, <*M*^2^>^44–46^. Accordingly, to obtain information on the preferential orbital and spin orientation and their correlation with the lattice distortion in the AFM phase of NTO, temperature-dependent Ni *L*_3,2_-edge XANES analyses were performed in the Dragon beamline BL-11A at the National Synchrotron Radiation Research Center (NSRRC) in Taiwan with electrically polarized X-rays aligned ‘nearly’ parallel (***E***// ***c***, θ = 70°) and perpendicular (***E***⊥ ***c***, θ = 0°) to NTO’s ***c***-axis, as shown in the top panel of Fig. 5(a). Typically, the absorption white lines at the Ni *L*_3,2_-edge depend strongly on the Ni 3*d*-3*d* and 2*p*-3*d* multiplet structures, corresponding to Coulomb and exchange interactions, the local crystal field (CF) and ligand-metal *pd* hybridization^47^. The bottom panel in Fig. 5(a) displays the temperature-dependent Ni *L*_3,2_-edge XLD (difference in the XANES features between ***E***// ***c*** and ***E***⊥ ***c***) of NTO. The alignment of the X-ray electric polarization field ***E***, both out-of-plane (***E***// ***c***) and in-plane (***E***⊥ ***c***), and Ni *L*_3,2_-edge XLD allow probing of the corresponding unoccupied Ni *e*_g_ 3*d*_3z_^2^_−r_^2^ and 3*d*_x_^2^_−y_^2^ orbitals (or the parallel and perpendicular projections onto the crystallographic ***c***-axis in the present NTO system), respectively^20,45,48^. The sign of the XLD spectra is negative in the paramagnetic regime (above 60 K), indicating that the Ni *e*_g_ holes preferentially occupy 3*d*_x_^2^_−y_^2^ (that is, preferential occupancy of the Ni *e*_g_ electrons in 3*d*_3z_^2^_−r_^2^) orbitals. However, upon cooling below *T*_SO_, the sign of the XLD spectra is reversed to positive, suggesting that the Ni *e*_*g*_ holes preferentially occupy the out-of-plane 3*d*_3z_^2^_−r_^2^ (that is, preferential occupancy of the Ni *e*_g_ electrons in 3*d*_x_^2^_−y_^2^) orbitals. This result is reproducible and consistent with similar temperature-dependent Ni *L*_3,2_-edge XANES and corresponding XLD measurements with electrically polarized X-rays on a different beamline [BL-20A at the NSRRC (not presented here)]. To elucidate this phenomenon, the integrated intensity of the area under the Ni *L*_3_-edge of XLD spectra (*A*_*L*3_, in the region from 848–858 eV) is plotted in Fig. 5(b), which clearly indicates the temperature dependency of the preferential electron occupancy in the Ni 3*d e*_g_ orbitals. Negative and positive values of *A*_*L*3_ at various temperatures demonstrate the preferential Ni *e*_g_ electron occupancy in the 3*d*_3z_^2^_−r_^2^ and 3*d*_x_^2^_−y_^2^ orbitals, respectively. Above *T*_SO_ (60 K), the negative *A*_*L*3_s are fairly independent of temperature, as shown in Fig. 5(b). The evolution of this anisotropic behavior can also be attributed to an additional local CF effect and ligand-metal *p-d* hybridization, which stabilize electron occupancy in the 3*d*_3z_^2^_−r_^2^ orbitals in the NTO. Although the origin of this CF effect and ligand-metal *p-d* hybridization is currently not considered, the distortion of the crystal structure is primarily responsible for the lowering of the energy of either the in-plane or out-of-plane *e*_g_ orbitals of transition metal oxides^45^. We believe that the inherent distortion of NiO_6_ octahedra in an environment of trigonal symmetry also plays an important role, just as VO_6_ octahedral distortion contributes importantly to orbit-lattice coupling in rare-earth vanadates^24,25^. Previous studies^6,14^ have revealed that the NiO_6_ octahedra in NTO are distorted and that the Ni-O-Ni bond distances and angles vary substantially among the three Ni sites (Fig. 1). This phenomenon may be responsible for additional CF and ligand-metal *p-d* hybridization effects and thereby stabilize Ni *e*_g_ electron occupancy in the 3*d*_3z_^2^_−r_^2^ orbitals above *T*_SO_ (60 K). Below *T*_SO_, the Ni *e*_*g*_ electrons preferentially occupy the in-plane 3*d*_x_^2^_−y_^2^ orbitals, as revealed by the positive *A*_*L*3_ values. Since NTO does not undergo any structural transition in the range of 11–300 K [Fig. 2(b)], the switching of the preferred orbital is not caused by a structural transition, unlike in lanthanum manganite^19^. Several reports have verified strong orbital-lattice coupling in rare-earth perovskites of the families *R*VO_3_^24,25^ and *R*TiO_3_ (*R* = La, Pr, Sm, Yb and Lu)^26^, in which VO_6_ octahedral distortion is most likely responsible for the orbital ordering. Therefore, the spin-orbit-lattice couplings that accompany local lattice distortion, owing to static disorder and the breakdown of lattice symmetry, certainly seem to favor (or strongly correlate with) preferential orbital occupancy and orbital ordering in various transition metal oxide systems^20,22–24,26,49^.

**Figure 6:**
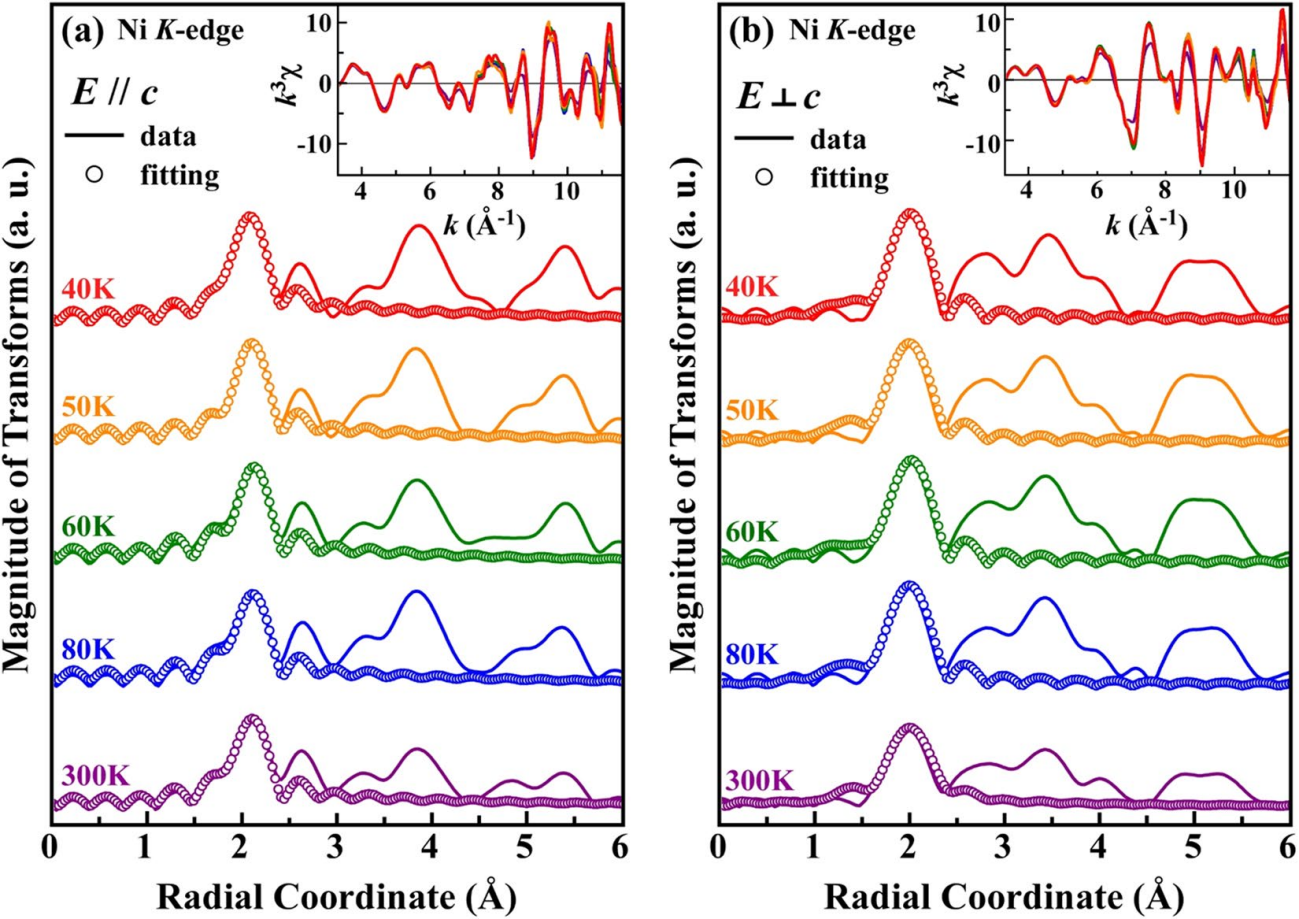
Magnitude of the FT spectra of the temperature-dependent Ni *K-*edge EXAFS for (**a**) ***E***// ***c*** and (**b**) ***E***⊥ ***c***, with corresponding *k*^3^-weighted *k*^3^ χ vs. *k* plots in the *k* range of 3.3–11.6 Å^−1^ shown in the respective insets. The first main FT feature (near *R* ~ 2 Å) corresponds to the mean NN Ni-O bond length within the NiO_6_ octahedra in NTO.

**Figure 7:**
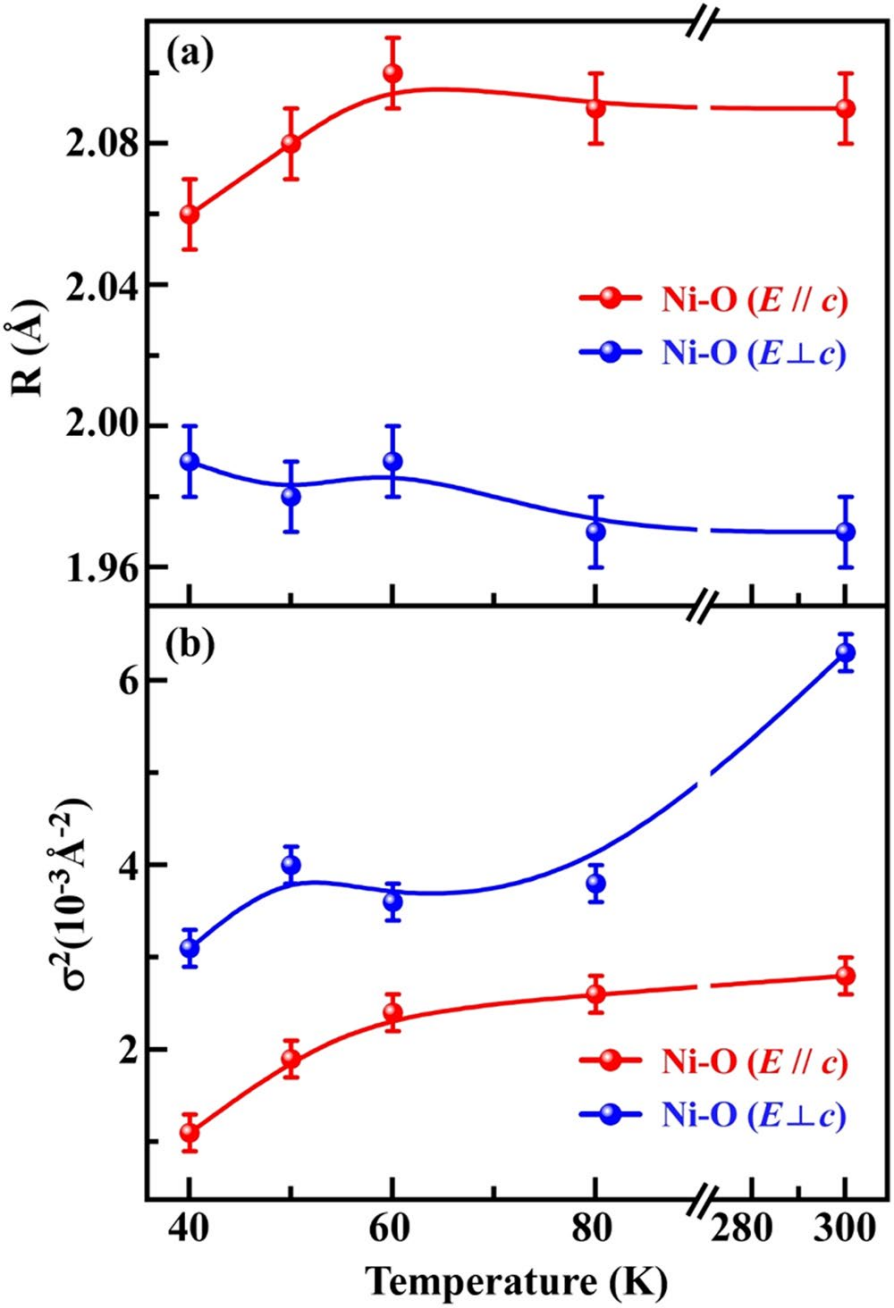
Variation in (**a**) NN Ni-O bond length and (**b**) DWF with temperature, obtained by fitting temperature-dependent Ni *K*-edge EXAFS spectra for *R* from 1.26 to 2.30 Å for ***E***// ***c*** and ***E***⊥ ***c*** polarizations in NTO.

To elucidate the correlation between the Ni 3*d* electron spin and the lattice, as well as the factors that drive the switching of the preferential orbital occupancy from out-of-plane to in-plane in the NTO single crystal below *T*_SO_, temperature-dependent Ni *K*-edge EXAFS measurements were performed with linearly polarized X-ray beams oriented along ***E***// ***c*** and ***E***⊥ ***c***. Figure 6(a,b) display the temperature-dependent Fourier transform (FT) plots (solid lines) of the Ni *K*-edge EXAFS spectra of the NTO single crystal at *θ* = 70° (***E***// ***c***) and *θ* = 0° (***E***⊥ ***c***), respectively, within a *k* range of 3.3–11.6 Å^−1^. The insets present corresponding EXAFS *k*^3^χ data. The first main feature of the FT plots of the Ni *K*-edge EXAFS spectra corresponds to the nearest-neighbor (NN) bond length of Ni-O in the NTO single crystal. We have analyzed the EXAFS region of the spectra by using the ATHENA and ARTHEMIS program packages^50^ to extract quantitative local information, such as the mean NN Ni-O bond length (*R*), its mean-squared fluctuation, called the Debye-Waller factor (DWF), and the coordination number (*N*) around the Ni sites in NTO for ***E***// ***c*** and ***E***⊥ ***c***. Figure 6(a,b) also show fitted plots (circles) of the temperature-dependent Ni *K*-edge FT for two polarization orientations, ***E***// ***c*** and ***E***⊥ ***c***, respectively. This work primarily focuses on the NN oxygen coordination number around the Ni sites, the variation in the NN Ni-O bond length, and the corresponding DWFs for different temperatures in NTO, for both the ***E***// ***c*** and ***E***⊥ ***c*** polarizations. Therefore, we fit the first main FT feature within an R range of 1.26–2.30 Å, which resembles the first shell around the Ni site. The goodness of fit, i.e., the *R*-*factor*, lies between 0.009 and 0.014 (see SI Table 2), which indicates an excellent match between the experimental data and the model system (constructed with a calculated NN oxygen coordination number and known lattice parameters^13^) used in fitting. Table 2 of the Supplementary Information and Fig. 7 present the fitted results for the first main FT feature, shown in Fig. 6(a,b). Notably, the coordination numbers of NN Ni-O for ***E***// ***c*** and ***E***⊥ ***c*** are calculated by considering the projections of the oxygen atoms in distorted NiO_6_ octahedra onto the ***c***-axis and the ***ab***-plane, yielding values of 2.4 and 3.6, respectively. In an octahedron where the two apical O atoms are parallel to the ***c***-axis and the remaining four O atoms are in the ***ab***-plane, the coordination numbers of NN O atoms for the ***E***// ***c*** and ***E***⊥ ***c*** polarizations are integers, namely, 2 and 4, respectively. However, in distorted octahedra as in NTO [shown in Fig. 1(a)], the projections of the O atoms onto the ***c***-axis and the ***ab***-plane are calculated based on the angles they make with the ***c***-axis. This results in fractional coordination numbers of NN O atoms for the ***E***// ***c*** and ***E***⊥ ***c*** polarizations.

Figure 7(a,b) plot the NN Ni-O bond lengths (weighted according to the angle between the electric polarization and bond direction) and DWFs, respectively, as functions of temperature. Clear anisotropy is observed in the mean Ni-O bond lengths, wherein the distorted NiO_6_ octahedra have a longer Ni-O bond length in the out-of-plane (***E***// ***c***) than in the in-plane (***E***⊥ ***c***) of NTO for the entire measured temperature range. The out-of-plane mean NN Ni-O bond length in the 60–300 K temperature range is fairly independent of temperature, but compression is observed upon cooling below *T*_SO_ (60 K). However, the in-plane mean NN Ni-O bond length is rather temperature-independent throughout the entire measured temperature range (40–300 K). Consistent with our earlier discussion of the preferential orbital occupancy of the Ni 3*d e*_g_ electrons in NTO, this anisotropy of the NN Ni-O bond lengths above *T*_SO_ may lead to additional CF and ligand-metal *p-d* hybridization effects, causing preferential Ni *e*_g_ electron occupancy in the 3*d*_3z_^2^_−r_^2^ orbitals in the paramagnetic (PM) region, as mentioned above. Below *T*_SO_, a tensile-like strain (compression of the Ni-O bond lengths projected out-of-plane) in the NiO_6_ octahedra can favor the preferential Ni *e*_g_ electron occupancy in the 3*d*_x_^2^_−y_^2^ orbitals^51^. Additionally, as shown in Fig. 7(b), the DWFs at Ni sites in NTO (for ***E*** ⊥ ***c***) deviate from their expected decreasing trend at and below *T*_SO_. The variation in the DWF can be understood with reference to the intensity of the first main FT feature (better views of the variation in intensity of the first Ni *K*-edge FT features for both polarizations are shown in Fig. S4 in the Supplementary Information), which is determined by the oxygen coordination number *N* and the DWF [σ^2^ (T)] at Ni sites^51,52^. Due to the absence of structural phase transitions in the measured temperature range [11–300 K, see Fig. 2(b)], it is fair to consider the oxygen coordination number, *N*, as independent of temperature. Therefore, a change in σ^2^ (T) is associated with a variation in FT intensity with temperature. The DWF has two components, σ^2^_s_ and σ^2^_v_ (T), which are associated with static disorder in atomic structure and thermal lattice vibration, respectively^52^. Since σ^2^_s_ is independent of temperature, thermal variation in the DWF is commonly associated with variation in σ^2^_v_ (T), which, according to the Einstein and Debye models^51,52^, decreases as temperature decreases. As expected, the DWFs under both polarizations decrease (the intensity of the first main FT feature increases, as shown in Fig. S4) as the temperature decreases from 300 to 80 K because of the σ^2^_v_ (T) component. Interestingly, below *T*_SO_, the intensity of the FT feature decreases as temperature decreases, causing the DWF to increase (for ***E***⊥ ***c***). This anomalous result clearly indicates that the static disorder component, σ^2^_s_, dominates the decrease in the σ^2^_v_ (T) component caused by the disorder produced by thermal vibration of the lattice. Therefore, the overall DWF, σ^2^ (T), increases meaningfully as temperature decreases below *T*_SO_ (60 K). The large static DWF, particularly in the ***ab***-plane (***E***⊥ ***c***), can be understood to arise from the large static distortion of the octahedral oxygen network around the Ni sites in NTO below *T*_SO_, which strongly influences the intensity of the FT feature associated with NN Ni-O bonds. The evolution of this static disorder can be understood with respect to phonon-assisted behavior^53,54^, which is related to a decrease in or breaking of the crystal lattice symmetry, especially in the ***ab***-plane of NTO. Infrared, Raman and time-domain THz spectroscopic studies have previously revealed phonon-softening modes in NTO in the ***ab***-plane below *T*_N_^16,18^, which were explained by spin-phonon coupling. Spin-phonon coupling induces local lattice distortion in the ***ab***-plane, and the corresponding local displacements modulate the magnetic interactions among the Ni ions in NTO, causing them to align along the ***c***-axis, thus resulting in low-temperature polarization^16^.

As stated earlier, the theoretical analysis of Wu *et al*.^14^ suggested that the magnetic dipole-dipole interaction is stronger than the spin-orbit coupling and is therefore responsible for the orientation of the Ni spins parallel to the ***c***-axis in NTO. Their calculations also revealed that the *J*_2_ interaction between the Ni spins (Ni_*II*_-Ni_*III*_) along the ***c***-axis is more strongly FM than the *J*_1_ interaction (Ni_*I*_-Ni_*II*_ in the ***ab***-plane). Consistent with these calculations, weak FM (~60 K) interactions are observed herein with the uncompensated component of the Ni spins aligned only along the ***c***-axis, as shown in the magnetization plots (Fig. 3) and the XMCD spectra [for H// ***c***, as shown in Fig. 4(b), at 59 K but not for H⊥ c, as shown in Fig. S3 of the Supplementary Information]. These interactions are followed by strong AFM (*T*_N_ ~ 52 K) interactions with the Ni spin axis aligned parallel to the ***c***-axis, as presented in the magnetization plots in Fig. 3(a). Notably, in NTO, the NiO_6_ octahedra are inherently distorted with *off*-centered Ni atoms above *T*_SO_, so the evolution of phonon-softening modes below *T*_SO_ in the lattice is expected to correlate with the (phonon-mediated) interaction between additionally *off*-centered Ni atoms with respect to O atoms in the NiO_6_ octahedra in the Ni_*I*_-Ni_*II*_ and Ni_*III*_-Te layers. Although EXAFS analysis does not reveal the exact Ni sites responsible for the phonon-softening modes, earlier studies^16,18^ have established that the phonon-softening modes in the ***ab***-plane of NTO are associated with the displacement of Ni_*I*_ atoms with respect to Ni_*II*_ and Ni_*III*_ atoms and the octahedral stretching mode, which involves the shifting of O atoms with respect to Ni atoms. Nevertheless, Ni displacement and octahedral stretching modes in the ***ab***-plane are evidently associated with the variation in Ni-O bond lengths. Figure 7(a) indicates that the NN Ni-O bond lengths are, on average, compressed along the ***c***-axis (***E***// ***c***) upon cooling below *T*_SO_. These variations in Ni-O bond lengths below *T*_SO_ may explain the additional *off*-centering/displacement of the Ni ions, which causes phonon softening below *T*_SO_. Typically, the process of *off*-centering of metal ions preserves the overall crystal symmetry, but it may change the structure factor, resulting in a modification of the intensity of the powder XRD feature^53^. Variation in the intensities of the powder XRD feature of NTO is observed in Fig. 2(b), as mentioned earlier, while the crystal structure is maintained throughout the range of measurement temperatures. The unusually high DWFs in the ***ab***-plane relative to those along the ***c***-axis throughout the specified temperature range further suggest anisotropic local structural ordering of NTO. In addition, the higher slope of the DWF as a function of temperature between 80 and 300 K suggests that the in-plane (***E***⊥ ***c***) NN Ni-O bond length is more sensitive to temperature than the out-of-plane (***E***// *c*) Ni-O bond length. This anomalous variation in DWFs at in-plane Ni sites and the compression of the apical projection (***E***// *c*) of Ni-O bonds, which are correlated with lattice-orbit coupling and occur in conjunction with spin-phonon coupling, stabilize the preferential occupancy of the Ni *e*_g_ electrons in the 3*d*_x_^2^_−y_^2^ orbitals in NTO below *T*_SO_. In contrast, the inherent distortion of NiO_6_ octahedra stabilizes the Ni 3*d*_3z_^2^_−r_^2^ orbital above *T*_SO_. Phonon-softening behavior and, consequently, anomalous DWF behavior in the ***ab***-plane below a transition temperature were recently observed in our study of a SrFeO_3−δ_ single crystal^45^. A detailed theoretical investigation must be conducted in the future to develop our understanding of the correlation between local lattice distortion and preferential orbital occupation and its effect on the spin-spin correlation function in the NTO single crystal.

In summary, the electronic/atomic structure, preferential Ni 3*d*-orbital occupation and magnetic properties of the NTO single crystal were elucidated through magnetization measurements and temperature-dependent Ni *L*_3,2_-edge XANES, XLD and XMCD and *K*-edge EXAFS spectroscopic techniques. The magnetization measurements reveal a transition from the PM phase at high temperature to the FM phase close to *T*_SO_ (~60 K), followed by an AFM transition close to *T*_N_ (~52 K). Consistent with theory, the FM interactions associated with Ni_*II*_-Ni_*III*_ spins (exchange interaction of *J*_2_) are responsible for most of the evolution of the XMCD spectra along the ***c***-axis close to *T*_SO_, whereas the corresponding signal is absent when a magnetic field is applied perpendicular to the ***c***-axis. The Ni *L*_3,2_-edge XLD spectra above *T*_SO_ reveal that Ni 3*d e*_g_ electrons preferentially occupy the out-of-plane 3*d*_3z_^2^_−r_^2^ orbital and switch to the in-plane 3*d*_x_^2^_−y_^2^ orbital at and below *T*_SO_. The inherent distortion of NiO_6_ octahedra and anisotropic NN Ni-O bond length stabilize the preferential Ni *e*_g_ electron occupation in the out-of-plane (3*d*_3z_^2^_−r_^2^) orbital above *T*_SO_. However, at and below *T*_SO_, a large static distortion of the NiO_6_ octahedra (tensile-like strain due to the compression of the Ni-O bond length projected out-of-plane) network around the Ni sites, associated with phonon-softening behavior, stabilizes the preferential Ni *e*_g_ electron occupation of the in-plane orbital (3*d*_x_^2^_−y_^2^). These strong anisotropic lattice-orbital and spin-phonon couplings are responsible for the evolution of anisotropic magnetic properties and orbital switching in the NTO single crystal.

## Methods

### Sample preparation and characterization

Single crystal NTO with a (001) plane was synthesized using the flux slow cooling method; details on the sample dimensions (~3 × 2 mm surface dimension) and preliminary characterizations can be found elsewhere^13^. A detailed structural study of NTO was carried out using temperature-dependent powder and room temperature single-crystal XRD. Magnetic and electronic properties were obtained from temperature-dependent magnetization, Ni *L*_3,2_-edge XANES, XMCD and XLD and Ni *K*-edge XANES and EXAFS measurements.

Single-crystal XRD measurements were made in 2*θ* and *θ* scan modes at room temperature on a four-circle X-ray diffractometer with the X-ray beam aligned with the (003) Bragg reflection of NTO. Cu *K*_α_ X-ray radiation with a spot size of ~2 mm in diameter was focused onto the sample surface. Temperature-dependent powder XRD was also performed at beamline BL-07A of the NSRRC in Taiwan. The energy of the X-ray beam was 14 keV (wavelength ~0.8856 Å), which was later transformed to the wavelength (1.5406 Å) corresponding to the energy of Cu *K*_α_ to maintain consistency with the single-crystal XRD data. Temperature-dependent FC and ZFC magnetization measurements were made using a Quantum Design superconducting quantum interference magnetometer in the temperature range of 2–300 K with external magnetic fields applied parallel and perpendicular to the crystallographic ***c***-axis of the NTO single crystal.

XANES measurements with a circularly polarized X-ray beam at the Ni *L*_3,2_-edge were carried out in beamline Dragon-BL-11A at the NSRRC (the X-ray beam spot size was ~0.3 × 0.1 mm on the sample surface), and the spectra were obtained in the total fluorescence yield (TFY) and total electron yield (TEY) modes after a magnetic field of H = 100 Oe was applied to NTO. By switching the direction of the magnetic field, NTO’s magnetization direction was made parallel (μ_+_) and antiparallel (μ_−_) to the helicity of the incident X-rays. The difference between the above two measurements [(μ_−_ − μ_+_)/(μ_−_ + μ_+_)] is referred to as XMCD. For the linearly polarized X-ray beam, Ni *L*_3,2_-edge measurements (in Dragon-BL-11A and BL-20A in the TEY mode at the NSRRC) and *K*-edge measurements (in BL-17C in the TFY mode at the NSRRC) were made at two angles of X-ray photon incidence (θ) with respect to the normal to the NTO surface, θ = 0^0^ (normal incidence, with the electric field ***E*** of the linearly polarized photons perpendicular to the ***c***-axis, ***E***⊥ ***c***) and θ = 70° (grazing incidence, with ***E*** almost parallel to the ***c***-axis, ***E***// ***c***). *L*_3,2_-edge XLD denotes the difference between the above two measurements for different θ (***E***// ***c*** − ***E***⊥ ***c***). The Ni *K*-edge EXAFS spectra were analyzed using the ATHENA and ARTHEMIS program packages^50^ to extract quantitative local information, such as the mean NN Ni-O bond length (*R*), its mean-squared fluctuation, called the DWF, and the coordination number (*N*) for ***E***// ***c*** and ***E***⊥ ***c***.

## Electronic supplementary material

Supplementary Information
