# Supplementary material for: Anisotropy in the magnetic interaction and lattice-orbital coupling of single crystal Ni3TeO6
Source: Sci Rep. 2018 Oct 25;8:15779. doi: 10.1038/s41598-018-33976-w (PMC6202349; doi:10.1038/s41598-018-33976-w)
Supplement: Supplementary file 1 — Supplementary Information [file 41598_2018_33976_MOESM1_ESM.pdf]

Supplementary Information

Anisotropy in the magnetic interaction and lattice-orbital coupling of single crystal Ni_3TeO_6

Anirudha Ghosh,[1] K.-H. Chen,[1] X.-S. Qiu,[1] S. H. Hsieh,[1] Y. C. Shao,[1] C. H. Du,[1] H. T. Wang,[2] Y. Y. Chin,[3] J. W. Chiou,[4] Sekhar C. Ray,[5] H. M. Tsai,[6] C. W. Pao,[6] H. J. Lin,[6] J. F. Lee,[6] Raman Sankar,[7] F. C. Chou,[7] W. F. Pong[1,*]

[1] Department of Physics, Tamkang University, Tamsui 251, Taiwan

[2] Department of Physics, National Tsinghua University, Hsinchu 300, Taiwan

[3] Department of Physics, National Chung Cheng University, Chiayi 621, Taiwan

[4] Department of Applied Physics, National University of Kaohsiung, Kaohsiung 811, Taiwan

[5] Department of Physics, University of South Africa, Johannesburg 1710, South Africa

[6] National Synchrotron Radiation Research Center, Hsinchu 300, Taiwan

[7] Center for Condensed Matter Science, National Taiwan University, Taipei 106, Taiwan

Table 1: Peak positions and corresponding (*h k l*) values of all peaks (between 2θ = 20°-72°), obtained from the powder X-ray diffraction (XRD) of powdered NTO, after being transformed to the wavelength of Cu K_α-X-ray.

2θ (degrees)	*h*	*k*	*l*
21.09	1	0	1
23.91	0	1	2
32.97	1	0	4
35.13	1	1	0
38.51	0	1	5
39.26	0	0	6
40.41	1	1	-3
41.34	0	2	1
42.96	2	0	2
48.95	0	2	4
50.81	1	0	7
53.11	2	0	5
53.69	1	1	-6
55.35	1	2	-1
56.66	2	1	-2
57.45	0	1	8
60.52	0	0	9
61.67	1	2	-4
63.03	3	0	0
63.28	0	2	7
65.30	2	1	-5
66.62	3	0	3
69.17	2	0	8
71.82	1	0	10

S1: Figure S1(a) and (b) show the temperature-dependent Ni *K*-edge XANES of NTO single crystal with two sample orientations with respect to the X-ray polarization direction, ***E***// ***c*** and ***E***⊥ ***c***, respectively, with corresponding derivative spectra presented at the bottom. For comparison, Ni *K*-edge XANES of powdered NiO and Ni_2O_3 samples, obtained at room temperature (300 K), are also shown. The spectra consist of a pre-edge region near 8333.0±0.2 eV, which corresponds to the Ni 1*s*→ 3*d* quadrupolar transition or a dipole-allowed transition from the 1*s* to the ligand-metal *pd* hybridized state; followed by the dipole-allowed 1*s*→ 4*p* main absorption near 8340.5±0.2 eV for both ***E***// ***c*** and ***E***⊥ ***c***, and a high-energy EXAFS region.[1] To evaluate the integrated intensity of the pre-edge peaks, a tail of the Gaussian function is subtracted as background[2] from the pre-edge region of the spectrum; the resultant spectra near the pre-edge for all samples are plotted in the insets in Figs. S1(a) and (b).

Ni^{2+} system like NiO has three ground state configurations $|3d^8>$, $|3d^9\underline{L}^1>$ and $|3d^{10}\underline{L}^2>$.[3] Similarly, a Ni-oxide system with Ni^{3+} ions has four ground state configurations $|3d^7>$, $|3d^8\underline{L}^1>$, $|3d^9\underline{L}^2>$ and $|3d^{10}\underline{L}^3>$, where $\underline{L}$ represents the oxygen 2*p* ligand-hole. Evidently, the first states $|3d^8>$ and $|3d^7>$ of the Ni^{2+} and Ni^{3+} compounds, respectively, are of pure *d* character, while the other states are *pd* hybridized states.[1] The pre-edge peak, shown clearly in the insets of Figs. S1(a) and (b), is attributable either to the dipole-allowed transition from core Ni 1*s* to one of the *pd* hybridized states (following the selection rule $\Delta l = \pm 1$) or the Ni 1*s*→ 3*d* quadrupole transition.[1] However, the intensity of the quadrupole transition is expected to be extremely lower than that of the dipole transition to the *pd* hybridized state.[4] The presented Ni *K*-edge XANES and corresponding derivative spectra include no observable peak shifts (corresponding to dipole-allowed Ni 1*s*→ 4*p* transition) between the NiO and Ni_2O_3. However, the integrated intensity of the pre-edge white-line (after background subtraction) between 8330.0 to 8335.5 eV differs substantially between NiO (0.05± 0.01) and Ni_2O_3 (0.12± 0.01). The intensity is higher in Ni_2O_3, implying that a greater contribution of the oxygen 2*p* ligand-hole in the *pd* hybridized valence state (neglecting the quadrupole transition).[1,5] Accordingly, following the arguments in Kuzmin *et al.*[1] and Garcia *et al.*[5], the hybridized ground state in NiO and Ni_2O_3 can be attributed mostly to $|3d^9\underline{L}^1>$ and $|3d^9\underline{L}^2>$, respectively. Therefore, based on the density of oxygen 2*p* ligand-holes states that are mixed with the unoccupied Ni 3*d* states, as revealed from the intensity of the Ni *K*-edge pre-edge peak white line, the valence state of Ni ions in NTO can be compared to that in NiO and Ni_2O_3.[6] In NTO, the integrated intensities of Ni *K* pre-edge white lines are 0.05± 0.01 and 0.06± 0.01 for ***E***// ***c*** and ***E***⊥ ***c***, respectively, which are similar to that in NiO, indicating that Ni is mostly in the 2+ valence state

in NTO. Also, no thermal variation in the pre-edge intensity is observed, indicating that the Ni valence state in NTO is stable and does not exhibit any variation/transition with temperature.

For better understanding of the unoccupied Ni 3*d* states, the Ni $L_{3,2}$-edge XANES spectra of powdered NTO, NiO and Ni_2O_3 at room temperature (300 K) were obtained. Figure S1(c) shows the Ni $L_{3,2}$-edge XANES spectra of powdered NTO and standard NiO and Ni_2O_3 for comparison. Two edge steps at the L_3 and L_2 edges, resulting from a transition to the continuum unoccupied states were subtracted, following an arctan-function, from the pre and post L_3- and L_2-edges of normalized data for all samples, and then the integrated intensities of the L_3- and L_2-edge were computed. The inset shows an expanded view of the Ni L_3-edge after the arctan-background was subtracted. The integrated intensities of $L_{3,2}$-edge white-line features of NTO, NiO and Ni_2O_3 are 21.05± 0.05, 20.08± 0.05, and 22.33± 0.05, respectively. The intensity of $L_{3,2}$-edge features in Ni_2O_3 is higher than that of those in NiO because of the dipole-allowed transition from Ni 2*p* to higher unoccupied 3*d* states in Ni_2O_3 [involving $|3d^7\rangle$, $|3d^8\underline{L}^1\rangle$ and $|3d^9\underline{L}^2\rangle$] than to the less unoccupied 3*d* states in NiO [involving $|3d^8\rangle$ and $|3d^9\underline{L}^1\rangle$]. The integrated intensity of the Ni $L_{3,2}$-edge white-line feature of NTO is similar to that of NiO, suggesting that the valence states (Ni^{2+}) of NiO and NTO are similar. Also, the line-shapes of the Ni $L_{3,2}$-edge absorption spectra (involving a dipole-allowed transition from the spin-orbit split Ni 2*p* to the Ni 3*d* level) of NiO and Ni_2O_3 are similar (and similar to that of NTO). Generally, the multiplet structures at the Ni L_3- and L_2-edge depend strongly on the Ni 3*d*-3*d* and 2*p*-3*d* Coulomb and exchange interactions, the local crystal field (CF) and ligand-metal *pd* hybridization.[7] However, the possible different *pd* mixings in NiO and Ni_2O_3 do not seem to have generated any observable difference in line-shape of their spectra. The Ni $L_{3,2}$-edge spectral line-shapes of NTO are similar to that of other Ni^{2+} oxide materials in the results of Hu *et al.*[8] and Abbate *et al.*[9], therefore supporting the claim that the dominant Ni valence state in NTO is Ni^{2+}. Detailed theoretical interpretation of Ni $L_{3,2}$- and *K*-edge spectra are behind the current interest of investigation and therefore left for future endeavor.

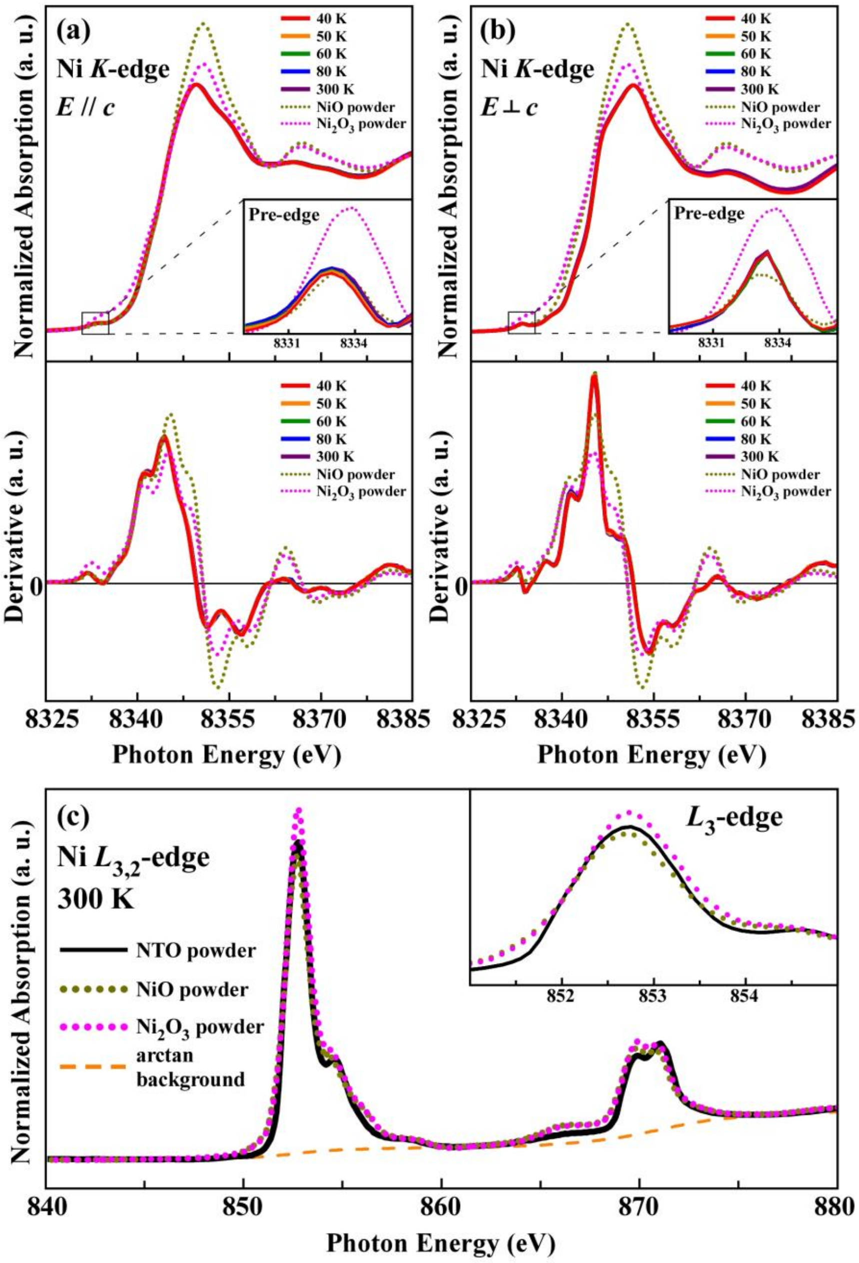

Figure S1: Temperature-dependent Ni *K*-edge XANES of NTO at two orientation of polarizations, (a) ***E***// ***c*** and (b) ***E***⊥ ***c***. Corresponding derivative spectra at both polarizations are presented at the bottom of respective XANES spectra. For comparison, Ni *K*-edge XANES of powdered NiO and Ni_2O_3 samples, measured at 300 K, are also shown. All spectra are normalized at pre-edge and post-edge regions. Insets of Figs. S1(a) and (b) show an expanded view of the pre-edge peak region of Ni *K*-edge XANES after background subtraction by the tail of Gaussian function. (c) Ni $L_{3,2}$-edge spectra of powdered NTO, NiO and Ni_2O_3 at 300 K along with the arctan-function (orange dashed curve) used as background. Inset shows the expanded view of the L_3-edge after subtraction of background with arctan-function.

S2: For further assurance of Ni^{2+} state in NTO, we have carried out temperature dependent Te *K*-edge XANES of NTO single crystals at two sample orientations with respect to the x-ray polarization direction, ***E***// ***c*** and ***E***⊥ ***c***, as shown in Figs. S2 (a) and (b), respectively and corresponding derivative spectra are shown at the respective bottom. The XANES threshold/peak positions (indicated by black dashed line) for both polarization of X-ray are similar, at 31825.0 ± 0.5 eV which are also independent of temperature, and in good agreement with the Te^{6+} state in $Te(OH)_6$ as reported by Grundler *et. al*. [10] Therefore assuming oxygen in 2- state, Ni should be in 2+ state to satisfy charge compensation in NTO. Also, since the Te *K*-edge XANES threshold/peak position does not vary within the measured temperature range (40-300 K), the valence state of Te (6+) and Ni (2+) are invariant in the aforementioned measurement temperature range.

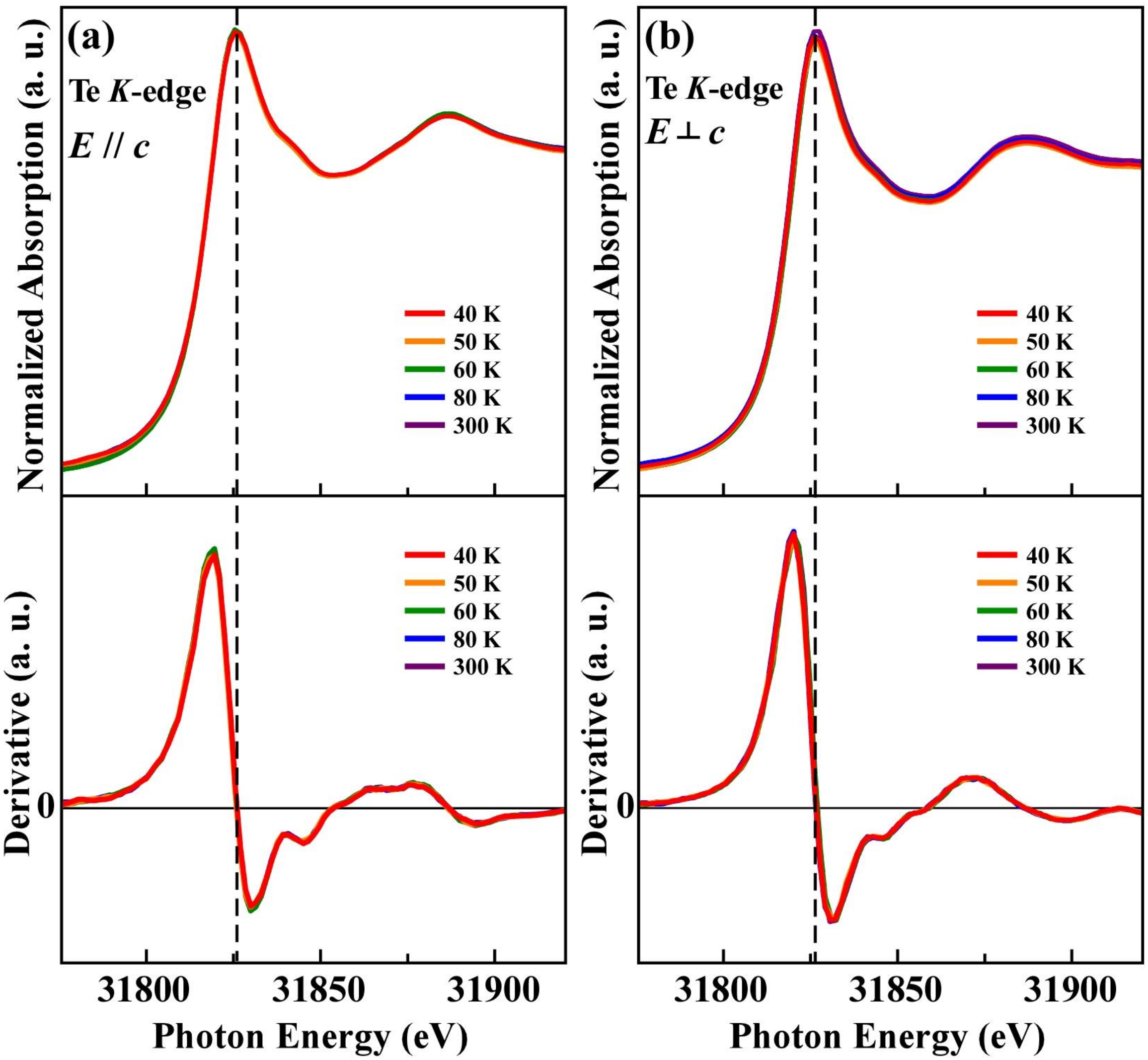

Figure S2: Temperature-dependent Te *K*-edge XANES of NTO at two orientation of polarizations, (a) ***E***// ***c*** and (b) ***E***⊥ ***c***. Corresponding derivative spectra at both polarizations are presented at the bottom of respective XANES spectra. The peak position of the Te *K*-edge XANES (shown by black dashed line) is 31825.0 ± 0.5 eV at both ***E***// ***c*** and ***E***⊥ ***c*** polarizations, which is in agreement with the XANES spectra for Te^{6+} state as reported by Grundler *et. al* [10] in their $Te(OH)_6$ sample.

S3: Temperature-dependent X-ray absorption spectra of NTO for circularly polarized X-ray, collected in total electron yield mode at a magnetic field of 100 Oe applied perpendicular to the ***c***-axis. The x-ray absorption data measured in fluorescence yield mode for H⊥ ***c*** (not shown) suffer self-absorption (since the fluorescence detector is perpendicular to the direction of the magnetic field and therefore normal to the sample surface) unlike that for H// ***c*** (now the fluorescence detector is grazing to the sample surface and therefore captures near-surface information with least self-absorption effect) data in Fig. 4, where the evolution of weak XMCD signal is consistent with the FM feature in magnetization measurements for H// ***c***. Two spectra (μ_- and μ_+) at a particular temperature have been collected at two opposite magnetic field direction and corresponding XMCD [$(\mu_- - \mu_+)/(\mu_- + \mu_+)$] spectra have been obtained from the difference of the two spectra. The temperature-dependent XMCD spectra do not have any feature at Ni $L_{3,2}$-edge, indicating that the Ni^{2+} spins are not aligned in ***ab***-plane at the onset of ferromagnetic interaction (~59 K).

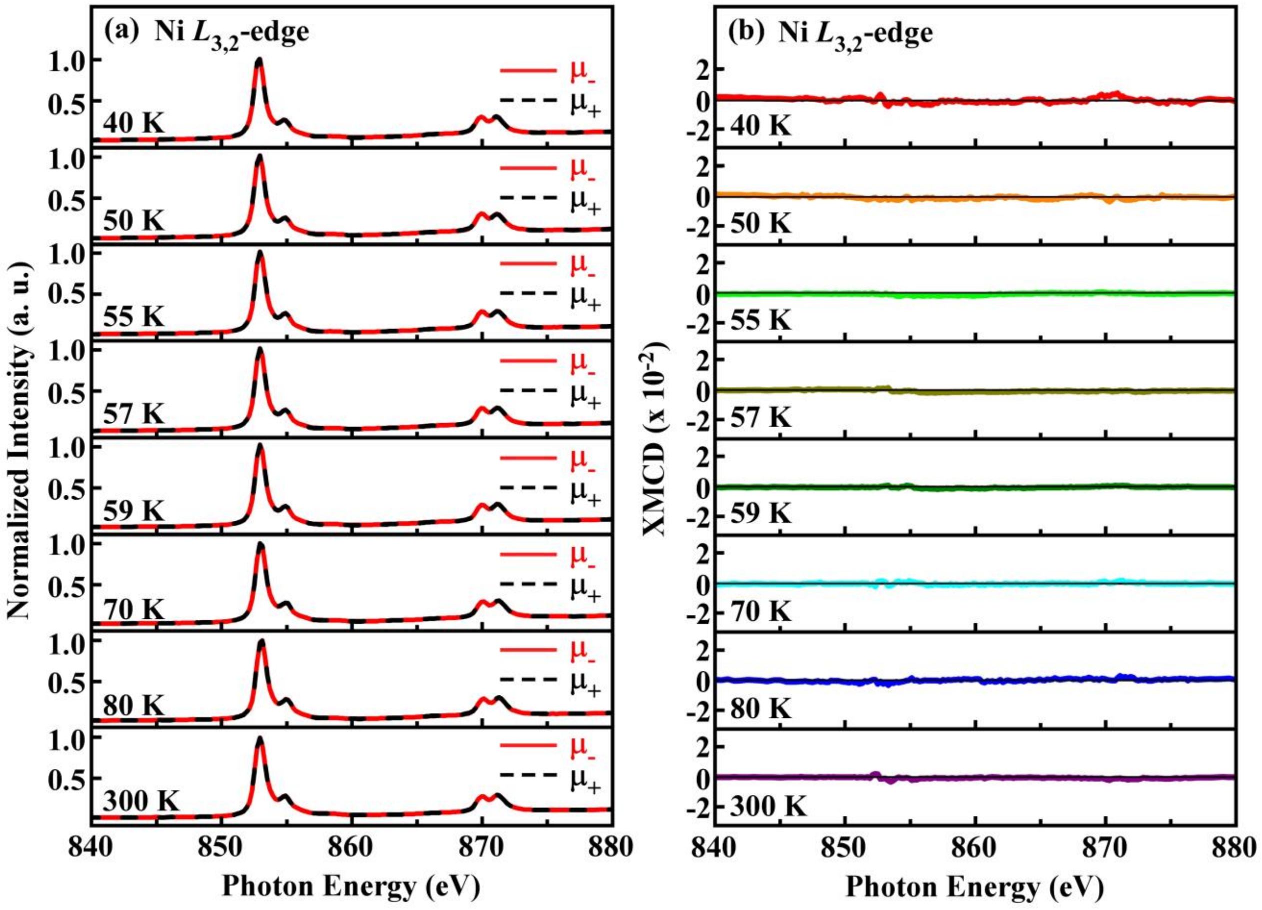

Figure S3: (a) Temperature-dependent Ni $L_{3,2}$-edge X-ray absorption spectra of NTO for circularly polarized X-ray, collected in total electron yield mode and corresponding (b) XMCD spectra.

Table 2: Best fit parameters obtained from the fitting of Ni *K*-edge EXAFS data in the *R*-space (the experimental FT was obtained for *k*-range 3.3-11.6 $Å^{-1}$) mode from 1.26 to 2.30 Å for ***E***// ***c***-axis and ***E***⊥ ***c***-axis (***ab***-plane). N_{ab}, σ^2_{ab}, and R_{ab} & N_c, σ^2_c, and R_c correspond to number of nearest-neighbor (NN) oxygen ions around central Ni ion, square of Debye-Waller factor for Ni-O, and mean NN Ni-O bond length in the ***ab***-plane and ***c***-axis, respectively. The distorted NiO_6 octahedra at all three Ni sites ensures contribution from all six oxygen atoms to N_c and N_{ab}. With the knowledge of Ni-O bond angles with respect to the ***c***-axis for all three Ni sites, we compute the projection of individual oxygen atoms along ***c***-axis and in ***ab***-plane and found $N_c = 2.4 \pm 0.1$ and $N_{ab} = 3.6 \pm 0.1$. The *R-factor*, which signifies the goodness of fit varies from 0.009 to 0.014 in the *ab*-plane (***E***⊥ ***c***) and ***c***-axis (***E***// ***c***). Typically, for excellent match between experimental data and model system, the *R-factor* should be below 0.02.[11] Therefore, the obtained *R-factors* which are below 0.02, resemble excellent fitting.

Temperature (K)	N_{ab}	N_c	σ_{ab}^2 (× 10^{-3} $Å^{-2}$)	σ_c^2 (× 10^{-3} $Å^{-2}$)	R_{ab} (Å)	R_c (Å)	*R*-factor (***ab***-plane)	*R*-factor (***c***-axis)
40	3.6 ± 0.1	2.4 ± 0.1	3.1 ± 0.2	1.1 ± 0.2	1.99 ± 0.01	2.06 ± 0.01	0.012	0.009
50	3.6 ± 0.1	2.4 ± 0.1	4.0 ± 0.2	1.9 ± 0.2	1.98 ± 0.01	2.08 ± 0.01	0.012	0.010
60	3.6 ± 0.1	2.4 ± 0.1	3.6 ± 0.2	2.4 ± 0.2	1.99 ± 0.01	2.10 ± 0.01	0.010	0.011
80	3.6 ± 0.1	2.4 ± 0.1	3.8 ± 0.2	2.6 ± 0.2	1.97 ± 0.01	2.09 ± 0.01	0.014	0.011
300	3.6 ± 0.1	2.4 ± 0.1	6.3 ± 0.2	2.8 ± 0.2	1.97 ± 0.01	2.09 ± 0.01	0.013	0.009

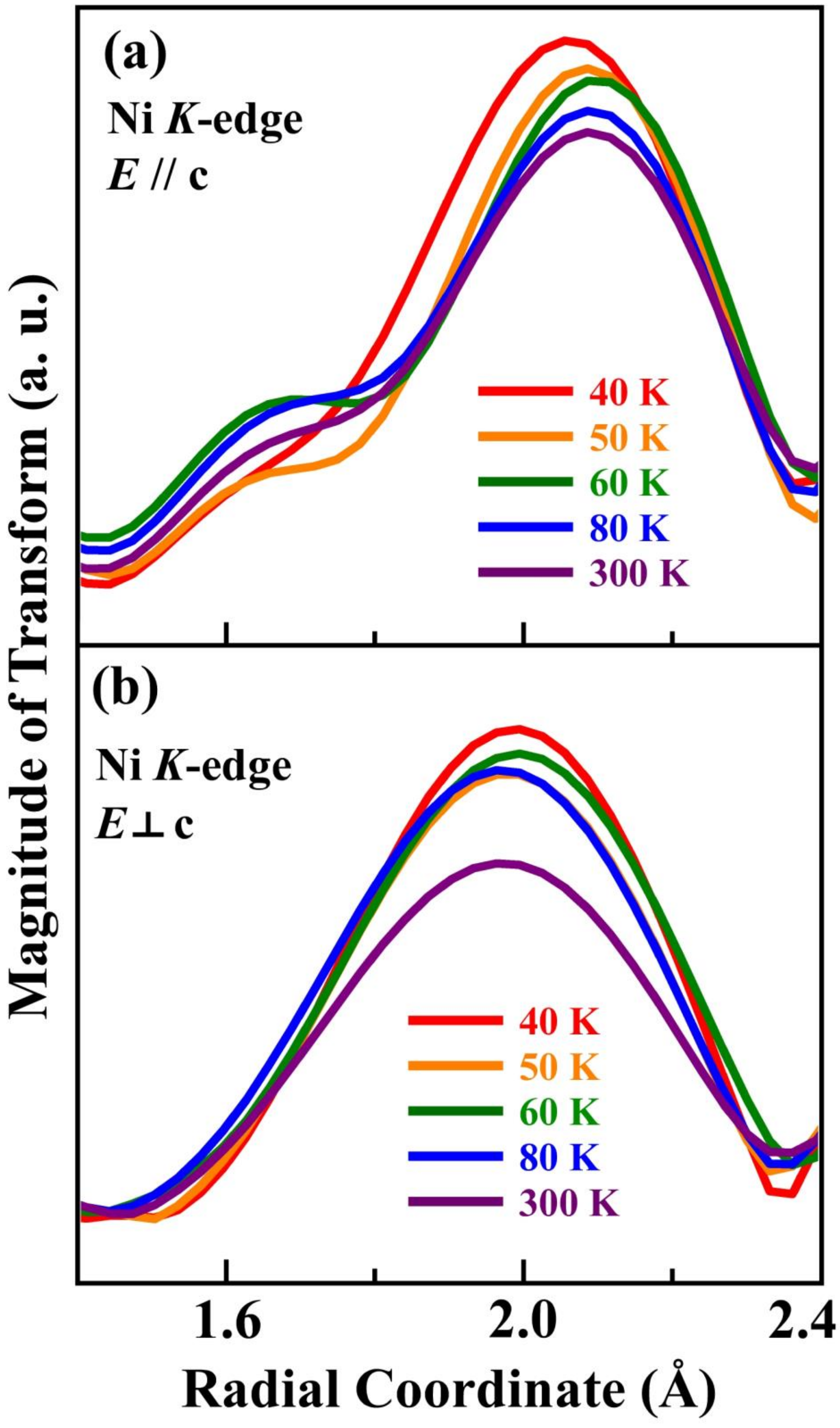

Figure S4: Temperature-dependent variation of the FT feature corresponding to the NN Ni-O bond distance of Ni *K*-edge EXAFS for (a) ***E***// ***c*** and (b) ***E***⊥ ***c***-axis.

References:

1. Kuzmin, A., Purans, J. & Rodionov, A. X-ray absorption spectroscopy study of the Ni K edge in magnetron-sputtered nickel oxide thin films. *J. Phys.: Condens. Matter* **9**, 6979 (1997).
2. Henderson, G. S., de Groot, F. M. F. & Moulton, B. J. A. X-ray Absorption Near-Edge Structure (XANES) Spectroscopy. *Rev. in Mineralogy & Geochemistry* **78**, 75 (2014).
3. van Veenendaal, M. A. & Sawatzky, G. A. Doping dependence of Ni 2p x-ray-absorption spectra of $M_xNi_{1-x}O$ (M =Li, Na). *Phys. Rev. B* **50**, 11326 (1994).
4. Dobrodey, N. V. & Luniakov. Y. V. Intensities of Electric Quadrupole Transitions in the X-Ray Spectra of Transition 3d-Metal Oxides. *Physica Scripta.* **50**, 19 (1994).
5. Garcia, J., Blasco, J. & Proietti, M. G. Analysis of the X-ray-absorption near-edge-structure spectra of $La_{1-x}Nd_xNiO_3$ and $LaNi_{1-x}Fe_xO_3$ perovskites at the nickel *K* edge. *Phys. Rev.* B **52,** 15823 (1995).
6. Wang, W.-C, Chen, Y. & Hu, T.-D. A Method of Data Processing for X-Ray *L*-edge Absorption. *Phys. Stat. Sol. (b)* **186**, 545 (1994).
7. van der Laan, G., Zaanen, J., Sawatzky, G. A., Karnatak, R. & Esteva, J.-M. Comparison of X-ray absorption with X-ray photoemission of nickel dihalides and NiO. *Phys. Rev. B* **33**, 4253 (1986).
8. Hu, Z. *et al.* Hole distribution between the Ni 3*d* and O 2*p* orbitals in $Nd_{2-x}Sr_xNiO_{4-\delta}$ *Phys. Rev. B* **61**, 3739 (2000).
9. Abbate, M. *et al.* Soft-X-ray-absorption studies of the location of extra charges induced by substitution in controlled-valence materials. *Phys. Rev. B* **44**, 5419 (1991).
10. Grundler, P. V. *et al.* Speciation of aqueous tellurium(IV) in hydrothermal solutions and vapors, and the role of oxidized tellurium species in Te transport and gold deposition *Geochimica et Cosmochimica Acta* **120**, 298 (2013).
11. Calvin, S. *XAFS for Everyone* (CRC Press, Taylor & Francis Group, 2012).
